# Very high gravity ethanol fermentation by flocculating yeast under redox potential-controlled conditions

**DOI:** 10.1186/1754-6834-5-61

**Published:** 2012-08-24

**Authors:** Chen-Guang Liu, Na Wang, Yen-Han Lin, Feng-Wu Bai

**Affiliations:** 1 School of Life Science and Biotechnology, Dalian University of Technology, Dalian, 116023, China; 2 Department of Chemical Engineering, University of Saskatchewan, Saskatoon, SK S7N5A9, Canada

**Keywords:** Flocculating yeast, Very high gravity, Ethanol fermentation, Redox potential

## Abstract

**Background:**

Very high gravity (VHG) fermentation using medium in excess of 250 g/L sugars for more than 15% (v) ethanol can save energy consumption, not only for ethanol distillation, but also for distillage treatment; however, stuck fermentation with prolonged fermentation time and more sugars unfermented is the biggest challenge. Controlling redox potential (ORP) during VHG fermentation benefits biomass accumulation and improvement of yeast cell viability that is affected by osmotic pressure and ethanol inhibition, enhancing ethanol productivity and yield, the most important techno-economic aspect of fuel ethanol production.

**Results:**

Batch fermentation was performed under different ORP conditions using the flocculating yeast and media containing glucose of 201 ± 3.1, 252 ± 2.9 and 298 ± 3.8 g/L. Compared with ethanol fermentation by non-flocculating yeast, different ORP profiles were observed with the flocculating yeast due to the morphological change associated with the flocculation of yeast cells. When ORP was controlled at −100 mV, ethanol fermentation with the high gravity (HG) media containing glucose of 201 ± 3.1 and 252 ± 2.9 g/L was completed at 32 and 56 h, respectively, producing 93.0 ± 1.3 and 120.0 ± 1.8 g/L ethanol, correspondingly. In contrast, there were 24.0 ± 0.4 and 17.0 ± 0.3 g/L glucose remained unfermented without ORP control. As high as 131.0 ± 1.8 g/L ethanol was produced at 72 h when ORP was controlled at −150 mV for the VHG fermentation with medium containing 298 ± 3.8 g/L glucose, since yeast cell viability was improved more significantly.

**Conclusions:**

No lag phase was observed during ethanol fermentation with the flocculating yeast, and the implementation of ORP control improved ethanol productivity and yield. When ORP was controlled at −150 mV, more reducing power was available for yeast cells to survive, which in turn improved their viability and VHG ethanol fermentation performance. On the other hand, controlling ORP at −100 mV stimulated yeast growth and enhanced ethanol production under the HG conditions. Moreover, the ORP profile detected during ethanol fermentation with the flocculating yeast was less fluctuated, indicating that yeast flocculation could attenuate the ORP fluctuation observed during ethanol fermentation with non-flocculating yeast.

## Background

Fuel ethanol that is renewable and environmentally friendly has been produced around the world as an alternative to fossil fuels [1]. However, high production cost makes fuel ethanol heavily dependent on preferential policies and governmental subsidies, especially in the United States and China where fuel ethanol is produced mainly from grain-based feedstocks [2]. Since feedstock and energy consumption are the major cost, lignocellulosic biomass, due to abundance and low cost, particularly agricultural residues, has been intensively studied for the production of fuel ethanol, but challenges are to be addressed to make such a process economically competitive [3]. Meanwhile, very high gravity (VHG) fermentation can significantly increase ethanol titer in the fermentation broth, which not only saves energy consumption for ethanol distillation, but also reduces waste distillage discharged from the distillation system, and thus has garnered great attention [2].

Apparently, ethanol-tolerant strains are prerequisite for more efficient ethanol production under VHG conditions in order to overcome stuck fermentation, in which significant sugars are present at the end of fermentation, and ethanol yield, the most important techno-economic aspect of fuel ethanol production, is compromised, correspondingly. Stuck fermentation results from severe ethanol inhibition in yeast cells [2]. When yeast cells flocculate, they can be retained and immobilized within fermentors for high cell density to improve ethanol productivity [4]. Moreover, ethanol tolerance of yeast flocs can be improved, since the morphological change caused by the flocculation consequently affects physiological functions of yeast cell membranes and intracellular metabolism [5,6], making yeast flocs more suitable for VHG fermentation. However, in situ monitoring the growth of yeast flocs and their fermentation performance under VHG conditions presents a challenge, since dissolved oxygen in the fermentation broth is undetectable under micro-oxygen conditions.

Redox potential (ORP) reflects electron activities during fermentations, which provides real-time information on the physiological status of cells [7]. Controlling ORP during fermentations has thus been developed as an effective method to enhance the production of desired metabolites such as 1,3-propanediol, citric acid and xylitol [8-10]. Recent studies have shown that for VHG ethanol fermentation with non-flocculating *Saccharomyces cerevisiae* subjected to ORP control, high ethanol productivity and yield could be achieved [11-13].

In this study, ORP profiles were monitored and analyzed for ethanol fermentation with the flocculating yeast. Furthermore, ORP control strategy was developed by controlling ORP at two different levels in order to improve ethanol productivity and yield for VHG ethanol fermentation with the flocculating yeast.

## Results and discussion

### Time courses of ethanol fermentation by the flocculating yeast

**Figure 1:**
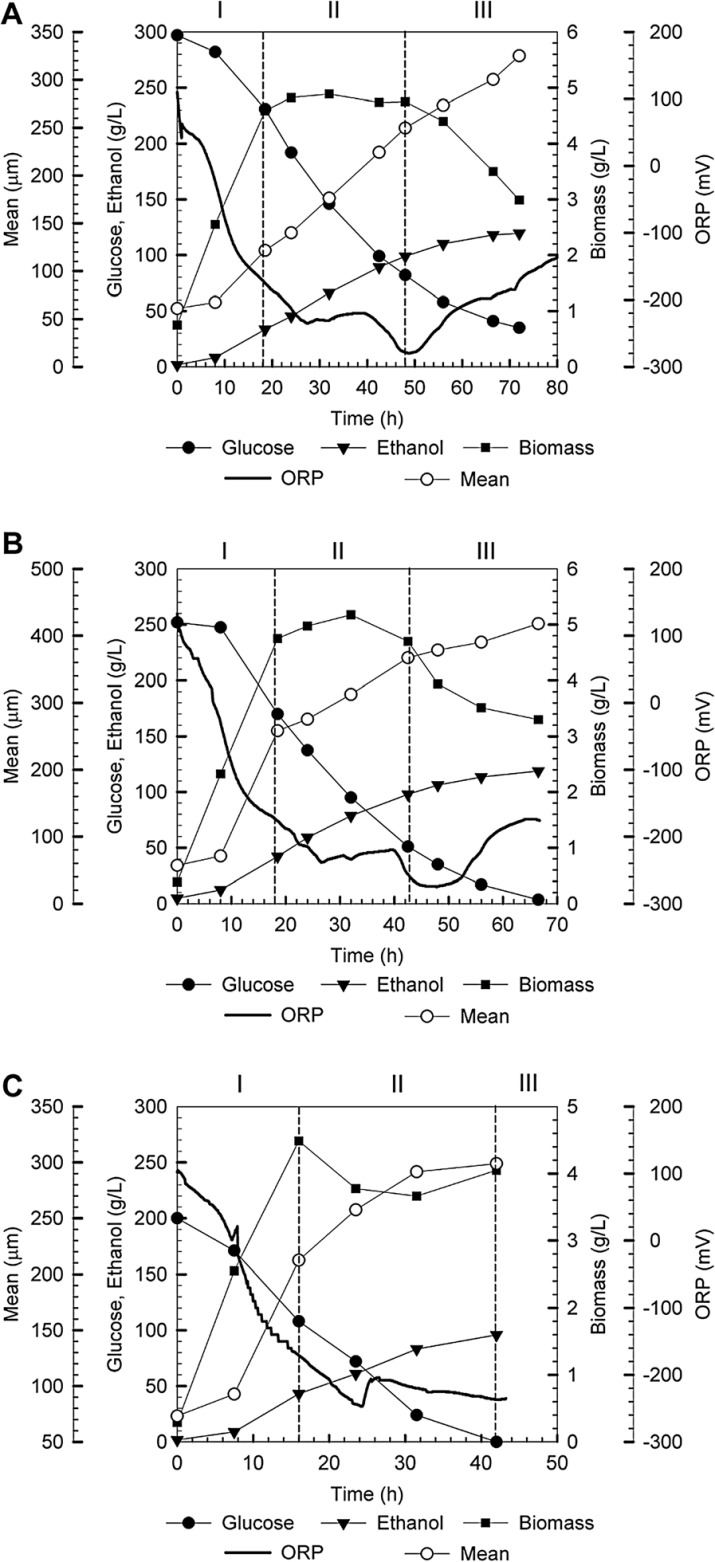
**Ethanol fermentation by flocculating yeast without ORP control.** Media containing glucose (g/L): 298 ± 3.8 (**A**), 252 ± 2.9 (**B**) and 201 ± 3.1 (**C**) Glucose consumption (solid cycle), ethanol production (solid triangle), biomass (solid square), ORP (bold line) and mean of yeast flocs (open cycle).

ORP affected the growth of non-flocculating *S. cerevisiae* and ethanol fermentation under VHG conditions [11]. Thus, time courses of glucose consumption, ethanol production, biomass accumulation, mean size of yeast floc and ORP were monitored for ethanol fermentation with the flocculating yeast, in which three regions were identified and illustrated in Figure 1.

When yeast flocs were inoculated into fresh media, vigorous growth quickly depleted dissolved oxygen in the media and fermentation reducing power outperformed oxidizing power, leading to a drastic decline of ORP from 110 mV to −220 mV in Region I, which was characterized by a logarithmic growth of yeast flocs. Yeast growth was then retarded due to ethanol accumulation and its inhibition effect, and a stationary phase was developed, correspondingly, which was characterized by a slow decrease of ORP till the lowest level of −280 mV in Region II. Near the end of fermentation, severe ethanol inhibition completely inhibited yeast growth, and in the meantime the lysis of yeast cells occurred, resulting in an increase of ORP.

Compared with ethanol fermentation with non-flocculating *S. cerevisiae*[11], time courses of ORP and other parameters detected for ethanol fermentation with the flocculating yeast were different. On the one hand, no lag phase was observed for the growth of yeast flocs, which indicated that yeast flocculation provided protection for yeast cells to adapt to the rapid change of physiological environment after inoculated into the HG and VHG media. On the other hand, fermentation time was prolonged under the same glucose concentration and biomass accumulation conditions, indicating that mass transfer limitation occurred with yeast flocs, which was consistent with the increase of the mean size of yeast floc distribution from about 50 μm detected at the beginning to about 250 μm detected near the end of the fermentation.

Figure 1 also illustrates effect of different glucose concentrations during ethanol fermentation by the flocculating yeast where no ORP control was implemented. Under 201 ± 3.1 g glucose/L conditions, it took 42 h to completely utilize glucose; whereas, it required 66 h for the case of 252 ± 2.9 g glucose/L. Although the fermentation time was prolonged to 72 h, there were 35.0 ± 3.2 g glucose/L remained unfermented under 298 ± 3.8 g glucose/L conditions. The unfermented glucose not only compromises ethanol yield that is calculated based on total sugars feeding into fermentation systems without deduction of residual sugars, but also imposes difficulty for the subsequent treatment of waste distillage. Therefore, corresponding strategies should be developed to address these issues.

### Effect of ORP control on ethanol fermentation by the flocculating yeast

**Figure 2:**
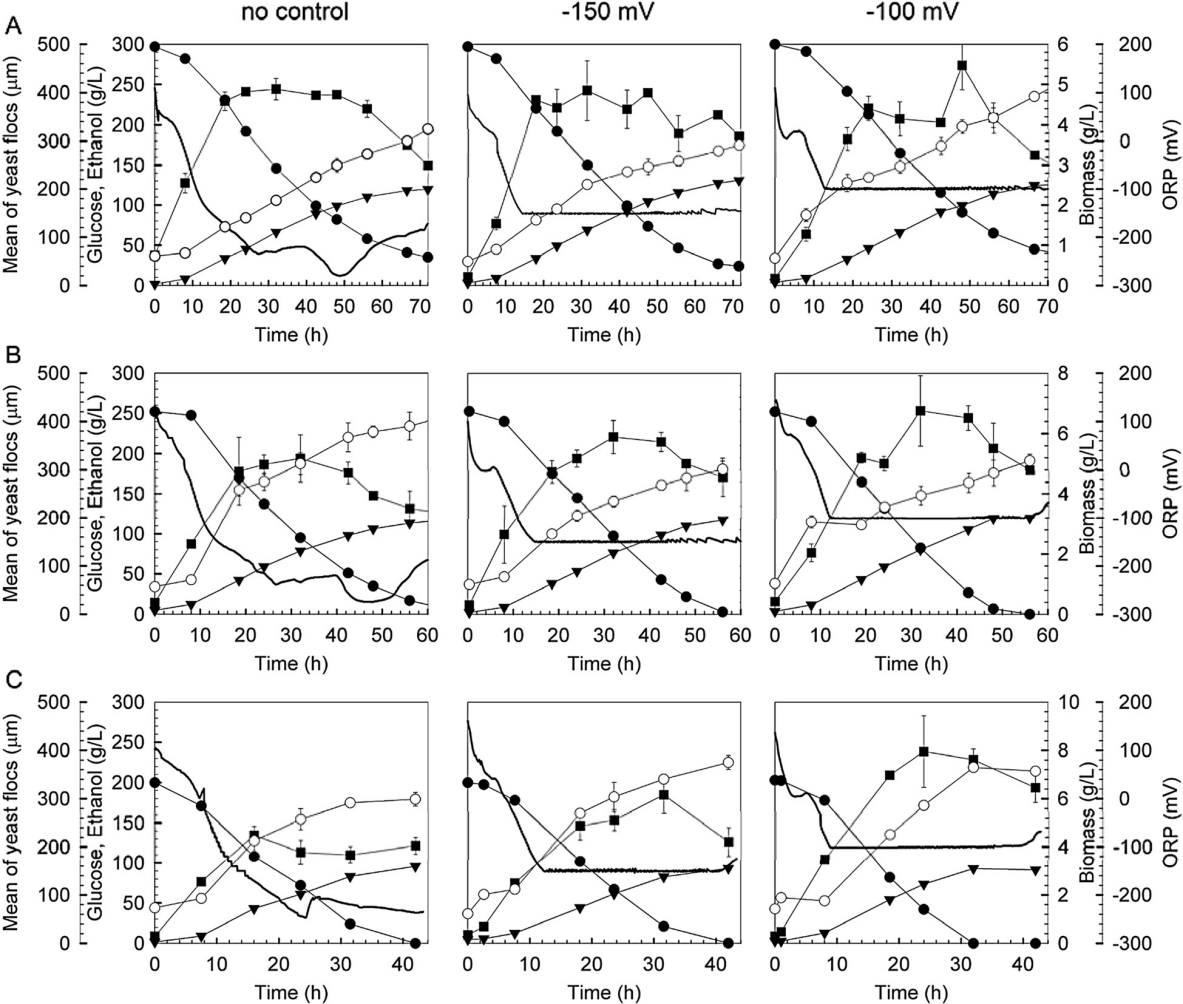
**Ethanol fermentation by flocculating yeast under ORP control conditions.** Media containing glucose (g/L): 298 ± 3.8 (**A**), 252 ± 2.9 (**B**) and 201 ± 3.1 (**C**). Glucose consumption (solid cycle), ethanol production (solid triangle), biomass (solid square), ORP (bold line) and mean of yeast flocs (open cycle).

Based on ORP profiles recorded in Figure 1 and previous studies on ethanol fermentation using non-flocculating yeast [11], two ORP levels at −100 and −150 mV were selected for ethanol fermentation by flocculating yeast under 201 ± 3.1, 252 ± 2.9 and 298 ± 3.8 g/L glucose conditions. Time courses of glucose consumption, ethanol production, biomass accumulation, mean size of yeast floc, and ORP were monitored and illustrated in Figure 2, and the experimental results were further summarized in Table 1.

**Table 1:** Summary of the experiment results

**Redox potential (mV)**	**Duration (h)**	**Glucose (g/L)**		**Ethanol (g/L)**		***Y***_**PS **_**(g/g)**	**Productivity (g/L/h)**	**Aeration (mL)**	Biomass^*^(g/L)	Yeast viability^*^(%)
		**Initial**	**Residue**	**Initial**	**Final**					
**~300 g (glucose)/L**										
−100			40.0 ± 0.2	4.1 ± 0.3	125 ± 1.7	0.466 ± 0.02	1.68 ± 0.02	1400	2.95 ± 0.04	49.25 ± 2.81
−150	72	298 ± 3.8	24.0 ± 1.1	3.2 ± 0.1	131 ± 1.8	0.467 ± 0.01	1.77 ± 0.02	200	3.13 ± 0.14	53.33 ± 4.96
No control			35.0 ± 3.2	1.8 ± 0.0	119 ± 1.5	0.449 ± 0.02	1.63 ± 0.02	0	2.98 ± 0.05	35.21 ± 4.21
**~250 g (glucose)/L**										
−100			0.2 ± 0.0	3.8 ± 0.3	120 ± 1.8	0.461 ± 0.03	2.07 ± 0.03	6550	4.80 ± 0.14	50.00 ± 4.68
−150			3.1 ± 0.1	2.5 ± 0.0	117 ± 2.2	0.461 ± 0.06	2.05 ± 0.04	1230	4.55 ± 0.64	59.09 ± 4.18
No control	56	252 ± 2.9	17.0 ± 0.3	4.9 ± 0.3	113 ± 1.4	0.462 ± 0.04	1.94 ± 0.02	0	3.51 ± 0.58	44.07 ± 2.06
**~200 g (glucose)/L**										
−100			0.0 ± 0.0	2.6 ± 0.0	93 ± 1.3	0.445 ± 0.02	2.82 ± 0.04	9220	6.45 ± 0.60	68.89 ± 6.22
−150	32	201 ± 3.1	21.0 ± 0.2	4.9 ± 0.3	83 ± 1.4	0.434 ± 0.02	2.43 ± 0.04	3120	4.20 ± 0.60	76.04 ± 5.85
No control			24.0 ± 0.4	1.8 ± 0.2	83 ± 1.5	0.463 ± 0.01	2.54 ± 0.05	0	3.05 ± 0.36	65.00 ± 13.3

^*^Data were collected at the end of fermentation.

In reference to ethanol fermentation without ORP control, more ethanol was produced when the ORP control strategy was applied. Since feedstock consumption is the major cost for fuel ethanol production [2], more ethanol production improved ethanol yield, and correspondingly saved feedstock consumption.

The highest ethanol concentration of 131.0 ± 1.8 g/L was obtained for ethanol fermentation with medium containing 298 ± 3.8 g/L glucose when ORP was controlled at −150 mV. Whereas, for ethanol fermentation with media containing glucose of 252 ± 2.9 and 201 ± 3.1 g/L, ethanol production was enhanced more significantly when ORP was maintained at −100 mV.

Ethanol production is attributed to both yeast cell viability and biomass accumulation. Yeast cell viability was improved more significantly when ORP was controlled at −150 mV, since more reducing power would favor the maintenance metabolism of yeast cells and correspondingly improve their viability [14,15], which might be the main reason for the enhancement of ethanol production under the VHG condition. On the other hand, more biomass was accumulated when ORP was controlled at −100 mV during ethanol fermentation with media containing 201 ± 3.1 and 252 ± 2.9 g/L glucose. The increase of biomass concentration was due to more air sparged into the fermentation system (Table 1), which stimulated yeast propagation, and consequently improved ethanol production.

Glucose was completely consumed for ethanol fermentation with media containing 201 ± 3.1 and 252 ± 2.9 g/L glucose when ORP was controlled at −100 mV. Although ethanol fermentation under 298 ± 3.8 g/L glucose conditions was improved when ORP was controlled at −150 mV, as high as 24.0 ± 1.1 g/L glucose was remained unfermented, which would be not acceptable from the point of view of industrial application. Hence, various fermentation strategies and process configurations such as tanks-in-series fermentation systems have been developed [16,17].

### Effect of yeast flocculation on ORP profile

**Figure 3:**
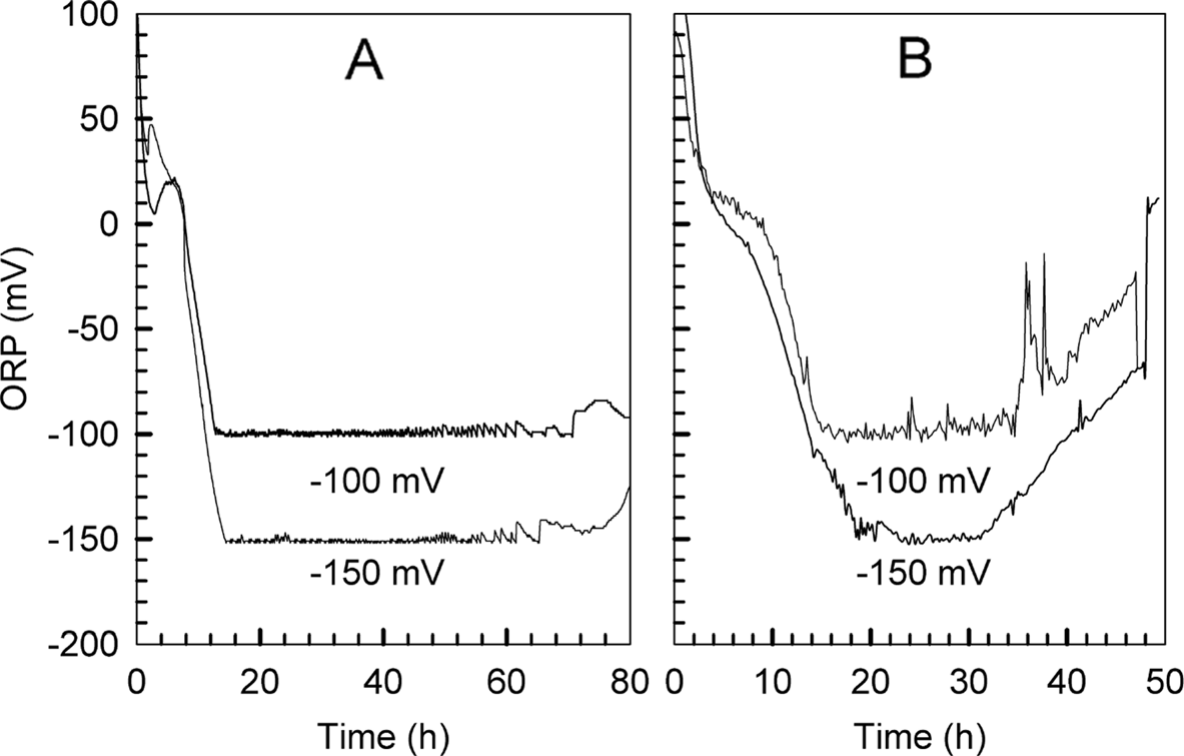
**ORP profiles of ethanol fermentation by flocculating yeast (A) and non-flocculating yeast (B).** Medium containing ~300 g/L glucose and ORP controlled at −100 and −150 mV, respectively. Data adapted from Lin et al. [11] for (B).

Compared with the bathtub ORP profile previously reported for VHG ethanol fermentation with *S. cerevisiae*[11], difference between the two ORP profiles (one with non-flocculating yeast, and another with flocculating yeast) was observed and illustrated in Figure 3.

Fluctuation in ORP during the stationary phase of non-flocculating yeasts was recorded. In contrast, ORP was relatively stable during ethanol fermentation with the flocculating yeast. We postulated that this difference might be due to the morphological change associated with the flocculation of yeast cells.

The flocculating yeast formed flocs during ethanol fermentation and the size of the yeast flocs increased as the fermentation proceeded (Figures 1 and 2). As more and more yeast cells flocculated together, a micro-environment was correspondingly developed among these cells. Yeast flocs are fragile in nature, and shear force induced by agitation breaks up larger flocs, and in the meantime smaller flocs re-flocculate and form large aggregates when they are in contact. As a result, metabolites occluded within yeast flocs and affecting ORP might release into the fermentation broth, and attenuate the ORP fluctuation observed in VHG ethanol fermentation with non-flocculating yeast. On the other hand, yeast flocculation also creates a physiological shelter to protect yeast cells from ethanol toxicity [18], as such the loss of yeast viability would be reduced (Table 1). It is noticed that there was no abrupt reduction of cell viability in the late stage of ethanol fermentation when the flocculating yeast was used; whereas, a sudden decline of yeast cell viability at the end of the stationary phase was reported during VHG ethanol fermentation when non-flocculating *S. cerevisiae* was employed [11].

## Conclusions

Compared to non-flocculating yeast, no lag phase was observed for growth and ethanol fermentation of the flocculating yeast under VHG conditions, which resulted in a drastic decline of ORP, and in the meantime stuck fermentation with prolonged fermentation time and significant glucose remained unfermented was observed due to mass transfer limitation associated with the flocculation of yeast cells. The ORP control provided a strategy for improving ethanol productivity and yield for the VHG fermentation system.

Ethanol fermentation performance is affected by biomass accumulation as well as yeast cell viability, and thus the ORP control impacts the fermentation system differently. With media containing glucose of 201 ± 3.1 and 252 ± 2.9 g/L, complete fermentation was observed at 32 and 56 h when the ORP was controlled at −100 mV, since yeast cell growth was stimulated by more oxygen supply through aeration. On the other hand, for ethanol fermentation with medium containing 298 ± 3.8 g/L glucose, as high as 131 ± 1.8 g/L ethanol was produced at 72 h when the ORP was controlled at −150 mV, because the lower ORP level with more reducing power available for maintaining metabolism of yeast cells improved their viability more significantly. Although the ORP control improved ethanol fermentation performance, residual glucose was still high for the VHG ethanol fermentation, which would be not acceptable from the view point of industrial application, and other bioprocess engineering strategies such as tanks-in-series systems that can alleviate ethanol inhibition in yeast cells should be developed and integrated with the ORP control.

When ORP control was applied, the ORP profile detected during ethanol fermentation with the flocculating yeast was more stable, compared with that observed in ethanol fermentation with non-flocculating yeast, indicating that the morphological change associated with the flocculation of yeast cells and micro-environment created within yeast flocs not only attenuated the flocculation of ORP, but also might prevent yeast lysis, which would benefit process control and optimization.

## Methods

### Yeast strain, media and ethanol fermentation

The flocculating yeast strain was developed at Dr. Bai’s lab at Dalian University of Technology [19]. This strain was cultured in the YPD medium composed of (g/L): yeast extract, 4; peptone, 3; glucose, 30; to the mid-log phase (around 18 h) in shake flasks, and inoculated into fermentor with 1 L working volume. The media contains (g/L): yeast extract, 6; peptone, 8; along with three different glucose concentrations: 201 ± 3.1, 252 ± 2.9 for high gravity fermentation, or 298 ± 3.8 for VHG fermentation. The inoculum level was 10% of the working volume. Temperature and pH were controlled at 30°C and 4.5, respectively. The agitation rate was set at 150 rpm for all runs.

### ORP control scheme

An autoclavable ORP electrode (model: Pt4805-DPAS-SC-K8S/225, Mettler Toledo, Switzerland) was inserted into the fermentor to monitor ORP, and the measured voltage signal was processed by a pH/ORP controller (model: Transmitter PC-3100, Suntex, Taiwan). When the measured ORP was lowered than the set point, a proper amount of filter-sterilized (0.2 μm nylon membrane) air was sparged into the fermentor to maintain ORP at designated levels: -100 or −150 mV. The amount of air was determined by the PID algorithm built-in in the pH/ORP controller.

### Analysis

A 4-mL fermentation broth was sampled every 6 or 8 h. The sample was centrifuged at 10000 × g for 5 min. The precipitate was washed twice with deionized water, dried at 85°C for 48 h, and weighted. The supernatant was used to quantify glucose and ethanol by HPLC (Model: Waters 1525) with an RI detector (Model: Waters 2414). An ion exclusion column (Model: Aminex HPX-87 H 300 × 7.8 mm, Bio-Rad, USA) was used to separate metabolites. The mobile phase consisted of 10 mM H_2_SO_4_, and the flow rate was set at 0.6 mL/min. The temperature for column and RI detector were set at 50°C. Yeast cell viability was determined as a ratio of viable to total cell numbers counted under microscope by methylene-violet staining procedure [20].

### Characterization of yeast flocs

A focused beam reflectance measurement system was used to monitor yeast flocs in situ during the fermentation, which were characterized by the statistic mean of the yeast floc distribution [21].

## Abbreviations

HG: High gravity; VHG: Very high gravity; ORP: Oxidation-reduction potential, also known as redox potential.

## Competing interests

The authors declare that they have no competing interests.

## Authors’ contributions

CGL, under the supervision of YHL and FWB, developed the research scheme and prepared the draft manuscript. NW carried out the experimental work. CGL, YHL and FWB involved data interpretation, result discussion, and manuscript revision. All authors agreed and approved the manuscript submission.

## Authors’ information

CGL completed his PhD study on very high gravity ethanol fermentation in 2011 at Dalian University of Technology (DUT), China, and is now working as a post-doctoral fellow at DUT. NW is a MSc graduate at DUT, working on ethanol fermentation kinetics of flocculating yeast. YHL is a Professor at the Department of Chemical and Biological Engineering, University of Saskatchewan, Canada. FWB is a Professor at DUT’s School of Life Science and Biotechnology. YHL and FWB co-supervised CGL's PhD study.
